# Microwave-assisted electro-peroxone process for rapid removal of high-concentration recalcitrant dye solutions

**DOI:** 10.1016/j.isci.2026.116810

**Published:** 2026-07-20

**Authors:** Seyed Masoud Razavi Mozafar Moghadam, Bita Ayati

**Affiliations:** 1 Faculty of Civil and Environmental Engineering, Tarbiat Modares University, P.O. box 14115-397, Tehran, Iran

**Keywords:** energy, kinetics, oxidation state, reactive species, Reactive Yellow 84, RY84

## Abstract

Azo dyes are persistent pollutants commonly found in industrial wastewaters. This study evaluated the microwave-electro-peroxone (MW-EP) process for degradation of Reactive Yellow 84 (RY84) and optimized the operating conditions, using the one-factor-at-a-time (OFAT) approach. The optimum conditions were an initial dye concentration of 300 mg/L, pH 6.5, current intensity of 400 mA, microwave power of 279 W, ozone flow rate of 0.288 g/h, and NaCl concentration of 0.035 M. Under these conditions, 91.36% dye removal was achieved within 17 min, with an energy consumption of 579.25 kWh/kg. Chemical oxygen demand (COD) and total organic carbon (TOC) removal efficiencies reached 75.3% and 55.8%, respectively. Scavenger experiments identified ȮH and ^1^O_2_ as the main reactive species, while a synergy index of 1.91 indicated a constructive interaction between MW irradiation and EP oxidation. The degradation followed first-order kinetics. These findings highlight the applicability of the MW-EP process for treating high-concentration dye-containing wastewater.

## Highlights

• The microwave electro-peroxone process rapidly degraded Reactive Yellow 84
• OFAT optimization achieved 91.36% dye decolorization efficiency within 17 min
• COD and TOC removal efficiencies reached 75.3% and 55.8%, respectively
• Hydroxyl and singlet oxygen radicals dominated degradation, showing strong synergy

## Graphical abstract

**Figure:**
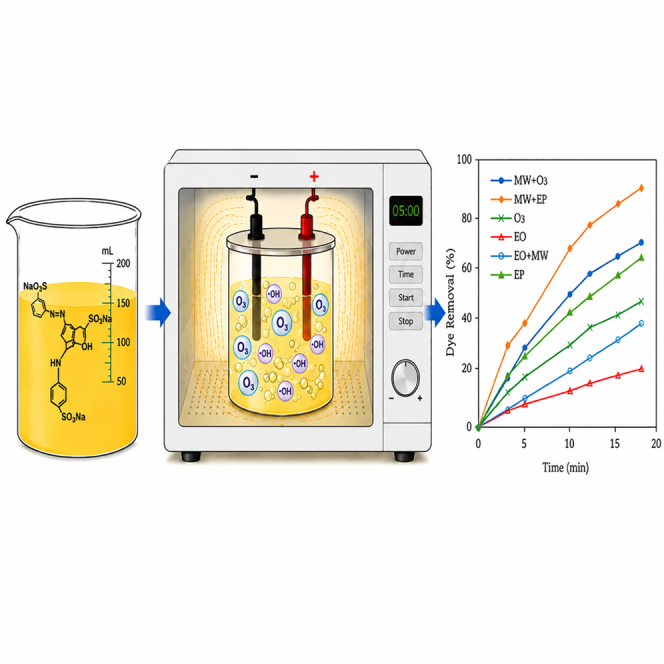

## Teaser

Chemical reaction kinetics; Pollution; Environmental assessment

## Introduction

The rapid expansion of industrial activities, particularly in the textile sector, has led to the discharge of large volumes of wastewater containing hazardous organic pollutants, posing severe threats to aquatic ecosystems and human health. Among these pollutants, azo dyes are of particular concern due to their complex aromatic structures, high solubility, and resistance to natural degradation.^1^ Recent studies have highlighted the critical need for sustainable water management strategies to mitigate the environmental footprint of such persistent contaminants.^2^ Furthermore, the limitations of conventional treatment methods have driven the search for advanced functional materials and technologies to enhance pollutant removal efficiency.^3^ Consequently, developing efficient, high-performance treatment processes for the mineralization of recalcitrant dyes remains a priority in environmental engineering.

Traditional treatment methods, including biological degradation, adsorption, and coagulation, often suffer from drawbacks such as slow kinetics, phase transfer of pollutants rather than degradation, and the generation of secondary sludge. To overcome these limitations, advanced oxidation processes (AOPs) have emerged as robust alternatives, which rely on the generation of highly reactive radicals to mineralize organic compounds into harmless end-products like CO_2_ and H_2_O. While various AOPs have been investigated, electrochemical advanced oxidation processes (EAOPs) have gained attention for their versatility and environmental compatibility.^4^^,^^5^ However, single AOPs often face challenges related to high energy consumption and limited mass transfer, necessitating the exploration of hybrid systems.^6^

To overcome the limitations of standalone processes, recent studies have shifted toward intensifying treatment via hybrid systems. A recent comprehensive review on Fenton and Fenton-like based AOPs^7^ underscores the critical need for such integrated configurations to overcome mass transfer resistances and maximize radical yield in complex matrices. Practically, the efficacy of this synergistic approach has been validated in investigations, such as the removal of Acid Red 33,^8^ where coupling oxidation mechanisms significantly enhanced the mineralization efficiency compared with individual treatments. Among electrochemical methods, the electro-peroxone (EP) process has shown remarkable efficacy. For instance, Ghalebizade and Ayati (2020)^9^ achieved 99% removal of Acid Orange 7 within 10 min, alongside removal efficiencies of 98% for chemical oxygen demand (COD) and 92% for total organic carbon (TOC). Efforts to further catalyze this process include the work of Naseri and Ayati (2024)^10^ who employed an egg-shell membrane-activated carbon/magnetite (EMAC/Fe_3_O_4_) nanocomposite, attaining pollutant, COD, and TOC removal efficiencies of 95.9%, 76.4%, and 54.3%, respectively, after 90 min. Similarly, Jenjaiwit et al. (2024)^11^ evaluated a continuous EP system, achieving rapid triclosan removal in 15 min, complete *E. coli* inactivation in 3 min, and 92.54% triclocarban removal after 120 min. However, EP efficiency remains pollutant dependent; Yang et al. (2021)^12^ reported a limited 64.87% removal efficiency for ibuprofen, highlighting the need for further intensification. While photo-assisted strategies utilizing non-TiO_2_-based photoanodes (Liu et al., 2025)^13^ or optimized UV-EP processes (Shokri et al., 2025)^14^ have been proposed, they are often restricted by poor light penetration in turbid textile effluents. In contrast, microwave (MW) irradiation offers a superior alternative through rapid volumetric heating. On the other hand, it has been recognized for its ability to accelerate chemical reactions through thermal and non-thermal effects (“hot spots”), significantly improving reaction kinetics. Recent advances in MW-assisted catalysis have demonstrated its potential to boost radical generation and reduce treatment times. Maheswari et al. (2022)^15^ demonstrated that MW treatment at 450 W could remove approximately 70% of COD in leachate within 10 min, outperforming conventional Fenton and ozonation. More recently, Wang et al. (2025)^16^ reported that an MW-enhanced Fenton-like process achieved 99.91% degradation and 87.17% mineralization of OTSA within just 6 min. Despite these advancements, the specific synergistic integration of MW irradiation with the EP system (MW-EP) for the treatment of recalcitrant reactive dyes remains underexplored.

To the best of our knowledge, this study presents the first comprehensive evaluation of the coupled MW-EP process for the degradation of Reactive Yellow 84 (RY84), a recalcitrant azo dye. The primary novelty lies in establishing the synergistic interaction between MW irradiation and the EP system to overcome the kinetic limitations of conventional electroporation. Unlike previous studies that often neglect the economic aspect, this work rigorously analyzes the specific energy consumption (SEC) alongside mineralization efficiency (TOC/COD) to propose a cost-effective treatment strategy. Furthermore, by employing radical scavengers, the dominant oxidative species are identified, clarifying how MW irradiation enhances radical generation beyond simple thermal heating. This research aims to provide a practical, energy-efficient framework for scaling up hybrid AOPs in treating complex industrial wastewaters.

## Results

### Parameter optimization

#### Dye concentration

**Figure 1:**
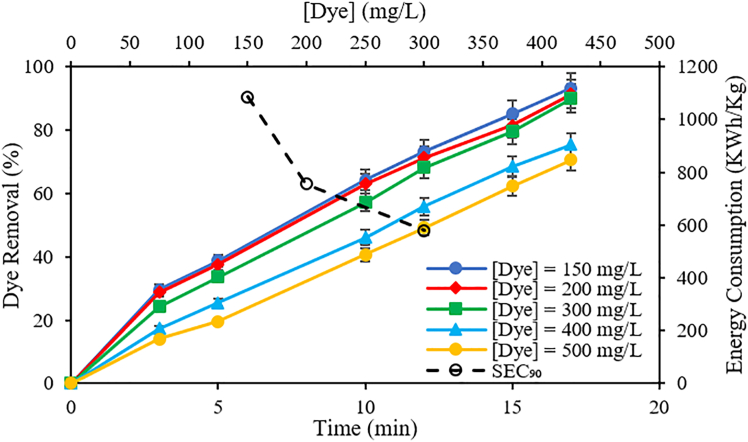
Effect of initial dye concentration on dye removal efficiency pH = 6.5, *p* = 279.04 W, I = 400 mA, [O_3_] = 0.288 g/h, [NaCl] = 0.035 M, and electrode distance (D) = 3 cm. Data are presented as mean ± SD (*n* = 3).

To determine the optimal operational range, the degradation of RY84 was evaluated at five distinct initial concentrations (150, 200, 300, 400, and 500 mg/L). As depicted in Figure 1, the removal efficiency followed a descending trend corresponding to the increased pollutant load—93.25%, 91.36%, 89.95%, 75.19%, and 70.63% within 17 min, respectively.

A critical analysis of these data reveals a transition between two distinct kinetic regimes. In the lower concentration range (150–300 mg/L), the system operates in a mass-transfer-controlled regime, where the abundance of hydroxyl radicals (ȮH) relative to dye molecules ensures high degradation rates (>90%). However, as the concentration exceeds the critical threshold of 300 mg/L, the system shifts toward a radical-limited regime. Because the generation rate of ȮH is constant (fixed electric current and MW power), the molar ratio of oxidant to pollutant decreases drastically. Theoretically, this creates a “scavenging effect” governed by competitive kinetics: the accumulation of reaction intermediates at high loads competes with the parent dye molecules for the available non-selective ȮH, thereby retarding the overall oxidation rate.^17^^,^^18^

From an energy perspective, the analysis of SEC provides a crucial insight into the economic feasibility. The SEC required to achieve 90% removal (SEC_90_) demonstrates an inverse relationship with concentration up to the optimal point. Increasing the concentration from 150 to 300 mg/L significantly reduced the energy consumption from 1,085.06 to 579.25 kWh/kg. This improvement is attributed to the enhanced collision probability between the generated radicals and the target pollutant molecules; at higher concentrations (up to 300 mg/L), the “wasted” energy consumed by parasitic reactions is minimized.

However, concentrations of 400 and 500 mg/L failed to reach the 90% removal threshold, indicating that the kinetic penalty of oxidant limitation outweighs the benefits of increased mass loading. Consequently, 300 mg/L was identified as the optimal concentration, representing the thermodynamic equilibrium where the driving force for mass transfer is maximized without saturating the oxidative capacity of the system.

### pH

**Figure 2:**
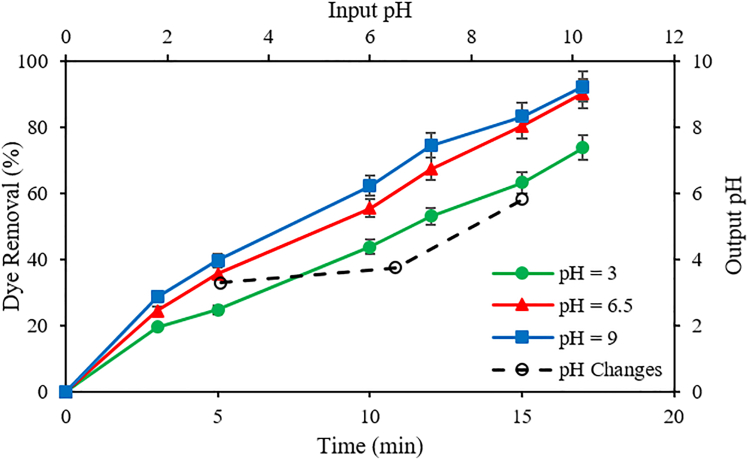
Effect of initial pH on dye removal efficiency [Dye] = 300 mg/L, *p* = 279.04 W, I = 400 mA, [O_3_] = 0.288 g/h, [NaCl] = 0.035 M, and electrode distance (D) = 3 cm. Data are presented as mean ± SD (*n* = 3).

To investigate the effect of initial pH on the dye removal efficiency, different pH values of 3, 6.5, and 9 were examined, and the results are presented in Figure 2. The pH variations showed that over time, the pH shifted toward acidic conditions. The removal efficiencies at the tested pH values were 73.68%, 90.06%, and 92.21%, respectively. From a mechanistic perspective, the ozonation process typically performs better under alkaline conditions. This is because the reaction of hydroxide ions (OH^−^) with ozone (O_3_) initiates a chain reaction, leading to the formation of hydroperoxide ions (HO_2_^−^), which are crucial precursors for ȮH generation (Equations 1 and 2). However, a critical analysis of the EP system suggests a trade-off: while high pH enhances O_3_ decomposition into radicals, it simultaneously destabilizes the electro-generated hydrogen peroxide (H_2_O_2_), causing its rapid self-decomposition before it can react with O_3_. Furthermore, at very high pH or excess concentrations, H_2_O_2_ species may act as scavengers, consuming the generated ȮH and reducing the overall oxidative capacity (Equation 3).^19^^,^^20^

(Equation 1) O_3_ + OH^−^→ HO_2_^−^ + O_2_

(Equation 2) O_3_ + HO_2_^−^→O˙H + O_2_ + O_2_^.−^

(Equation 3) O˙H + H_2_O_2_→ HO_2_^.^ + H_2_O

Another possible mechanism is that the primary form of H_2_O_2_, or its conjugate form, reacts with O_3_ to generate ȮH (Equation 4), which subsequently react with pollutants in aqueous solutions.^21^

(Equation 4) 2HO_2_^−^ + H_2_O + 2O_3_→ ȮH + 2OH^−^ + 2HO_2_^.−^ + 3O_2_

Under acidic conditions (pH 3), the moderate efficiency is attributed to the high stability of electro-generated H_2_O_2_ and the direct attack of molecular ozone, though the production rate of ȮH is kinetically slower than that under neutral/alkaline conditions. The complexity of these competing mechanisms—radical generation vs. scavenging and oxidant stability—has been corroborated by other studies.^22^ Based on the results, pH 6.5 was selected as the optimal value. This selection is justified not only by the high removal efficiency but also by the operational advantage of operating at near-natural pH, which eliminates the costs associated with pH adjustment and minimizes chemical consumption.

### MW power

The degradation of RY84 (300 mg/L) was investigated at four distinct MW power levels (161.32, 279.04, 354.27, and 527.37 W). A critical constraint was applied: the reaction was terminated once the solution reached the boiling point. This approach was chosen to isolate the influence of MW-induced thermal activation on bond cleavage and radical generation, limiting the complex stochastic effects of cavitation that dominate post-boiling regimes.^23^

**Figure 3:**
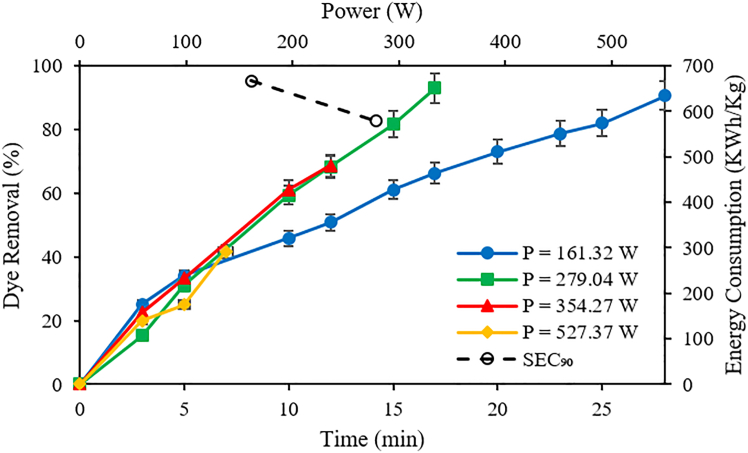
Effect of microwave power on dye removal efficiency [Dye] = 300 mg/L, pH = 6.5, I = 400 mA, [O_3_] = 0.288 g/h, [NaCl] = 0.035 M, and electrode distance (D) = 3 cm. Data are presented as mean ± SD (*n* = 3).

As illustrated in Figure 3, the removal efficiency exhibited a non-monotonic trend with increasing power: 90.62% (161.32 W), 92.96% (279.04 W), 68.67% (354.27 W), and 41.77% (527.37 W). To elucidate the underlying mechanism—specifically distinguishing between thermal and non-thermal MW effects—control experiments were conducted using an external ice-cooling jacket. Negligible degradation under isothermal conditions confirmed that non-thermal effects alone are insufficient for activation in this specific MW-EP system. This implies that the system is thermally driven, where MW energy provides the necessary activation energy (E_a_) to accelerate reaction kinetics.^24^ Furthermore, we cannot use reflux or cooling system for simple MWs.

From a theoretical standpoint, the sharp decline in efficiency at higher powers (354 and 527.37 W) represents a kinetic trade-off. While higher power input significantly increases the reaction rate constant (k) by rapidly elevating the temperature (Arrhenius equation), it disproportionately reduces the reaction time (t) required to reach the boiling point. At 527.37 W, the rapid heating rate shortens the effective contact time between the oxidant and pollutant to such an extent that it creates a temporal limitation. The reaction time becomes insufficient for complete mineralization, outweighing the benefits of the increased rate constant.^25^^,^^26^ Conversely, at 279.04 W, an optimal balance is struck where the temperature is high enough to facilitate rapid radical attacks, yet the heating duration allows sufficient residence time for the degradation reactions to proceed.

The energy efficiency at the 90% removal threshold is depicted in Figure 3. The power levels of 354 and 527 W failed to reach this threshold. Between the successful candidates, 279.04 W demonstrated superior energy performance with an SEC of 579.25 kWh/kg, relative to 667.49 kWh/kg at 161.32 W. Consequently, 279.04 W was selected as the optimal power, representing the synergistic peak of thermodynamic activation and energy efficiency.

### Current density

**Figure 4:**
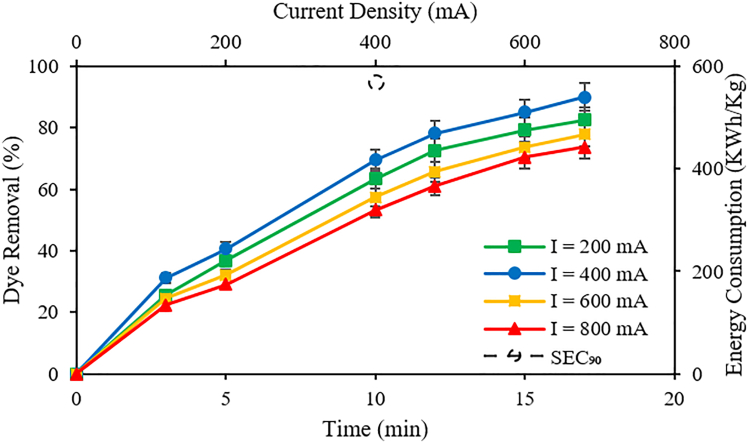
Effect of current density on dye removal efficiency [Dye] = 300 mg/L, pH = 6.5, *p* = 279.04 W, [O_3_] = 0.288 g/h, [NaCl] = 0.035 M, and electrode distance (D) = 3 cm. Data are presented as mean ± SD (*n* = 3).

Following the optimization of MW power, the influence of applied current intensity on the degradation efficiency was investigated at levels of 200, 400, 600, and 800 mA. As illustrated in Figure 4, the removal efficiencies were 82.61%, 90.01%, 77.81%, and 73.66%, respectively. Increasing the current from 200 to 400 mA significantly enhanced the process efficiency. This improvement is attributed to accelerated electrochemical kinetics, specifically the 2-electron reduction of dissolved oxygen at the cathode, leading to higher *in situ* H_2_O_2_ generation and subsequent production. However, a transition occurred beyond 400 mA, where further increases in current led to a decline in efficiency. This shift implies the dominance of parasitic side reactions over pollutant degradation.^27^^,^^28^^,^^29^ At excessive current densities, mass transfer limitations become critical, as the rate of O_3_ dissolution and reaction becomes the limiting factor compared with the rapid electrochemical generation of species, leading to unreacted oxidants.^22^^,^^30^ Simultaneously, high current densities create a sufficiently negative cathode potential to reduce generated H_2_O_2_ to water (Equation 5), instead of reducing oxygen. Additionally, the excess radicals trigger self-scavenging effects (Equation 6), forming hydroperoxyl radicals (H $\dot{O}$_2_) with lower oxidation potential.^27^^,^^28^^,^^29^

(Equation 5) HO_2_^−^ + H_2_O + 2e^−^→ 3OH^−^

(Equation 6) H_2_0_2_ + O˙H→ HO˙_2_ + H_2_O

The SEC_90_ for the 400 mA current was calculated at 579.25 kWh/kg (indicated by the dashed line). Because other current levels failed to reach the 90% removal threshold or consumed excessive energy due to parasitic reactions, 400 mA was selected as the optimal current intensity.

### O_3_ dosage

**Figure 5:**
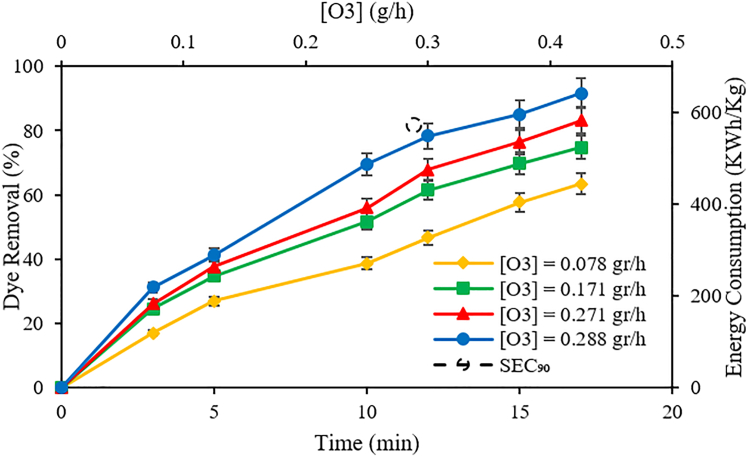
Effect of ozone concentration on dye removal efficiency [Dye] = 300 mg/L, pH = 6.5, *p* = 279.04 W, I = 400 mA, [NaCl] = 0.035 M, and electrode distance (D) = 3 cm. Data are presented as mean ± SD (*n* = 3).

Following the optimization of electrical parameters, the influence of the O_3_ mass flow rate on degradation performance was evaluated at 0.078, 0.171, 0.271, and 0.288 g/h. As depicted in Figure 5, the removal efficiencies were 63.91%, 74.81%, 83.11%, and 91.63%, respectively. The results demonstrate a direct correlation between O_3_ dosage and removal efficiency. At lower dosages (e.g., 0.078 g/h), the process is limited by the insufficient molar ratio of O_3_ to electro-generated H_2_O_2_, hindering the effective generation of ȮH via the peroxone reaction. Increasing the O_3_ dosage to 0.288 g/h significantly enhanced the degradation rate. This improvement is mechanistically attributed to the enhanced driving force for mass transfer; a higher inlet O_3_ flow elevates the partial pressure in the gas phase, thereby increasing the saturation concentration at the gas-liquid interface according to Henry’s Law. This reinforced concentration gradient accelerates O_3_ dissolution and its subsequent reaction with H_2_O_2_ and the cathode surface, intensifying the production of reactive oxygen species (ROS). However, the non-linear improvement observed between intermediate dosages suggests competing phenomena. While higher O_3_ input generally favors radical generation, excessive dosage can lead to O_3_ slip (unreacted O_3_ escaping in the off-gas) due to limited gas residence time or radical scavenging, where excess O_3_ consumes ȮH (Equation 7) rather than participating in pollutant oxidation.^28^ Therefore, a balance must be struck between dosage and utilization.

(Equation 7) O_3_ + OH^−^→ HO_2_^−^ + O_2_

In terms of energy efficiency, the 90% removal threshold—defined as the target for practical application—was achieved exclusively at the 0.288 g/h dosage, corresponding to an SEC of 579.25 kWh/kg (indicated in Figure 5). Because lower dosages failed to meet the removal target and higher dosages would incur diminishing returns due to mass transfer limitations, 0.288 g/h was selected as the optimal O_3_ dosage. These findings align with those of Chen et al.^1^ who reported an effective O_3_ dosage range of 0.2–0.3 g/h for similar AOPs.

### Electrolyte type

**Figure 6:**
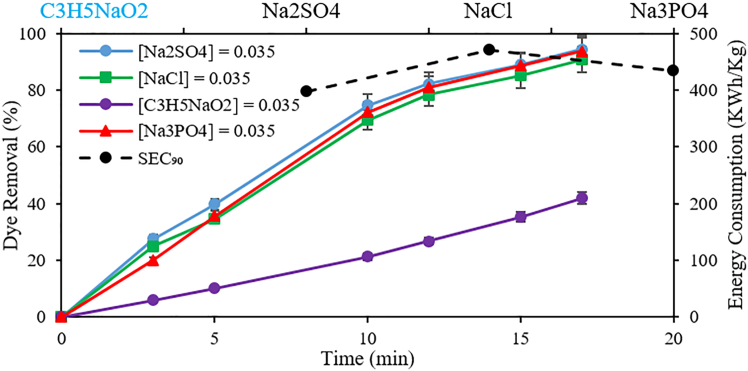
Effect of electrolyte type on dye removal efficiency [Dye] = 300 mg/L, pH = 6.5, *p* = 279.04 W, I = 400 mA, [O_3_] = 0.288 g/h, and electrode distance (D) = 3 cm. Data are presented as mean ± SD (*n* = 3).

The effect of different electrolytes on the current between electrodes was subsequently investigated. The dye removal efficiencies using sodium chloride (NaCl), sodium sulfate (Na_2_SO_4_), sodium phosphate (Na_3_PO_4_), and sodium propionate (C_3_H_5_NaO_2_) as an organic electrolyte were 90.7%, 94.36%, 93.74%, and 41.84%, respectively (Figure 6). The results indicated a sharp contrast between inorganic and organic electrolytes. The significantly lower efficiency in the presence of sodium propionate is attributed to the scavenging effect of the organic anion. Propionate ions compete with the target pollutant for ȮH, thereby consuming the oxidative capacity of the system without contributing to dye degradation. Conversely, inorganic electrolytes provided high removal rates.^31^ Although Na_2_SO_4_ showed marginally higher efficiency (94.36%) and lower SEC (397.86 kWh/kg for 90% removal) than NaCl (470.19 kWh/kg), NaCl was selected as the optimal electrolyte. This decision was based on its cost effectiveness, high conductivity, and, more importantly, its ability to generate secondary oxidants (active chlorine species), which provide a synergistic oxidation pathway alongside radical attack (further discussed in the following section).

### Electrolyte concentration

**Figure 7:**
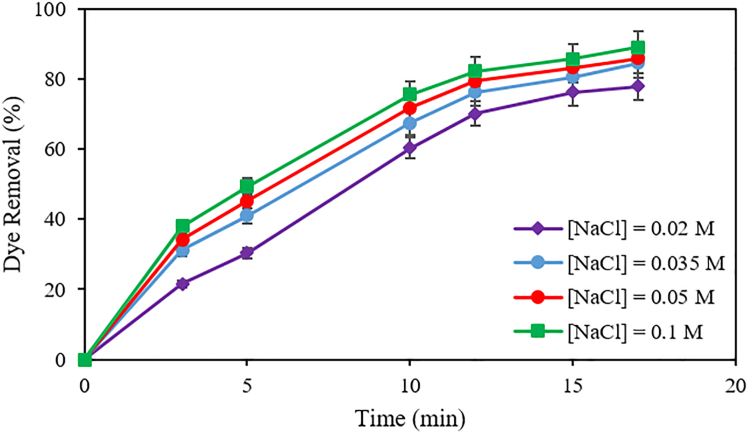
Effect of electrolyte concentration on dye removal efficiency [Dye] = 300 mg/L, pH = 6.5, *p* = 279.04 W, I = 400 mA, [O_3_] = 0.288 g/h, and electrode distance (D) = 3 cm. Data are presented as mean ± SD (*n* = 3).

Following the selection of NaCl, the effect of its concentrations—0.02, 0.035, 0.05, and 0.1 M—on process performance was investigated (Figure 7); the removal efficiencies were 77.96%, 84.63%, 89.07%, and 89.87%, respectively.

Increasing the NaCl concentration up to 0.05 M enhanced the removal efficiency. This improvement is primarily due to increased solution conductivity, which lowers the cell voltage at a constant current, thereby facilitating electron transfer and reducing energy consumption. However, beyond 0.05 M, the efficiency plateaued. It is crucial to note that chloride ions (Cl^−^) are not merely passive charge carriers; they undergo anodic oxidation to generate dissolved chlorine (Cl_2_), which hydrolyzes to form hypochlorous acid (HClO) and hypochlorite ions (ClO^−^) (Equations 8, 9, and 10).

(Equation 8) 2Cl^−^ → Cl_2_ + 2e^−^

(Equation 9) Cl_2_ + H_2_O ⇌ HClO + H^+^ + Cl^−^

(Equation 10) HClO ⇌ ClO^−^ + H^+^

These active chlorine species act as secondary oxidants, facilitating indirect oxidation of the dye molecules. While this supports degradation, excessive chloride concentration can be detrimental. High levels of Cl^−^ can act as scavengers for the more potent ȮH (E_0_ = 2.8V), converting them into chlorine radicals with lower oxidation potential, thus limiting further efficiency gains.^32^ Consequently, to balance conductivity, secondary oxidant generation, and radical scavenging, 0.035 M was selected as the optimal concentration.

### Electrode distance

**Figure 8:**
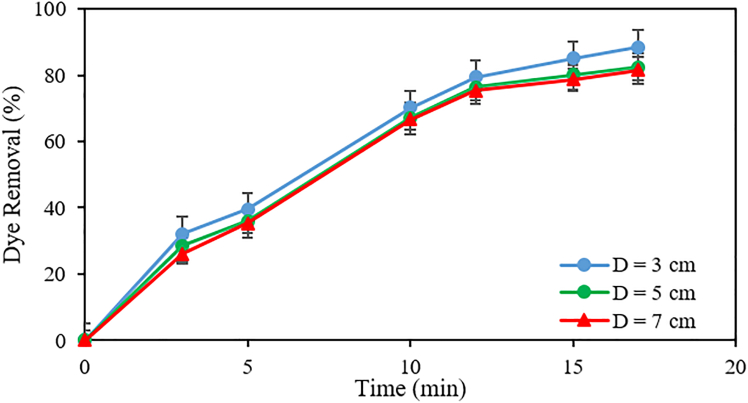
Effect of inter-electrode distances on dye removal efficiency [Dye] = 300 mg/L, pH = 6.5, *p* = 279.04 W, I = 400 mA, [O_3_] = 0.288 g/h, and [NaCl] = 0.035M. Data are presented as mean ± SD (*n* = 3).

The influence of inter-electrode distance on the dye removal efficiency was investigated at intervals of 3, 5, and 7 cm. As depicted in Figure 8, the removal efficiencies were recorded at 88.5%, 82.36%, and 81.46%, respectively. The results indicated a decrease in efficiency as the distance increased. This decline is primarily attributed to the increased ohmic resistance (IR drop) across the electrolyte solution, which hinders the ion transport and reduces the effective current available for electrochemical reactions. However, the reduction in efficiency was not drastic, suggesting that non-electrochemical mechanisms—specifically O_3_ oxidation and MW irradiation effects—also play a dominant role in the degradation process.^33^ Consequently, an inter-electrode distance of 3 cm was selected as the optimal value to minimize energy loss and maximize removal efficiency.

### Role of individual and combined processes

**Figure 9:**
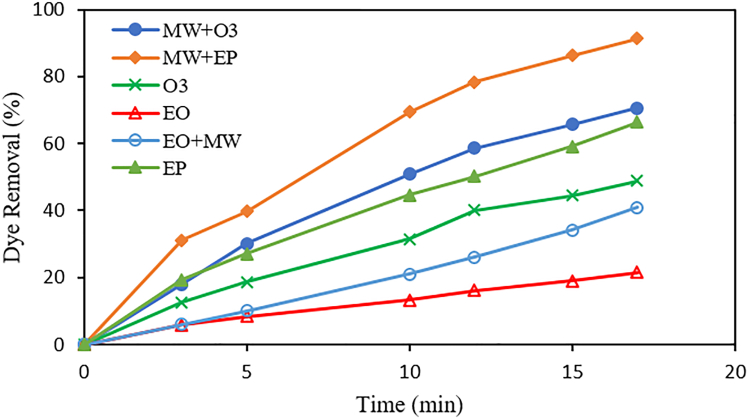
Effect of the processes used on dye removal efficiency [Dye] = 300 mg/L, pH = 6.5, *p* = 279.04 W, I = 400 mA, [O_3_] = 0.288 g/h, [NaCl] = 0.035 M, and electrode distance (D) = 3 cm.

To investigate the synergistic effect (SE) of the combined system, the role of each individual process in the MW-EP system was examined (Figure 9). The comparison indicated that each individual method has its specific limitations. The removal efficiency for the individual electrolysis system was only 21.5%, which can be attributed to its limited production of reactive species.^32^ Combining electrolysis with MW irradiation increased the removal efficiency to 40.87%, likely due to the enhanced temperature and stimulation of surface reactions in the presence of MWs.^34^ In the individual ozonation process, 48.82% of the pollutant was removed. O_3_ alone exhibits better performance because of its strong oxidizing ability, but its low solubility and short half-life limit the maximum removal efficiency.^35^ Meanwhile, the combination of MW and O_3_ showed a higher removal efficiency of 70.55%, indicating the SE between the thermal and non-thermal effects of MWs and the advanced oxidation capability of O_3_.^36^ For the individual EP process, the removal efficiencies were 66.42% at 17 min and 92% at 40 min. In contrast, the MW-EP process achieved the highest removal efficiency among all tested methods, reaching 91.36% at 17 min.

The results of the SE analysis indicated that the SE value for the MW-EP combined process was approximately 1.91, clearly greater than one, demonstrating a significant synergistic interaction between the two processes. This finding shows that the reaction rate in the combined process is considerably faster than the sum of the reaction rates of the individual MW and EP processes. This synergy can be attributed to several concurrent factors. First, the MW field induces rapid and uniform heating of the medium, while its non-thermal effects facilitate the generation of reactive radical species such as ȮH.^34^ Second, the EP process, through the simultaneous production of H_2_O_2_ and intermediate species along with promoted anodic oxidation, enables O_3_ activation and ȮH generation. When combined with MW irradiation, this substantially enhances the production of oxidizing species.^37^ Furthermore, the synergistic effect arising from the interaction of these two processes includes improved mass transfer, better mixing, and increased oxidizing capacity of the system. This combination not only reduces the reaction time but also enhances the pollutant removal efficiency.^38^

The use of EP as an AOP demonstrated that this method exhibits a reasonable efficiency when applied individually, as the combination of electrolysis and ozonation enables the simultaneous generation of ȮH and ROS. However, when the MW-EP process was employed, the highest removal efficiency was achieved. This indicates a triple synergistic effect among MW irradiation, electrochemical radical generation, and O_3_-induced oxidation.

### Investigation of the effect of reactive species on dye removal

**Figure 10:**
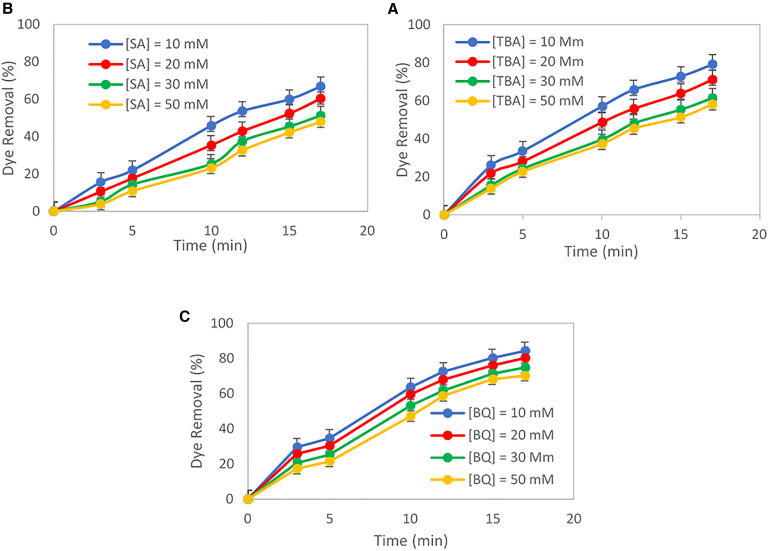
Effect of different scavenger concentrations on dye removal (A) Effect of different TBA concentrations. (B) Effect of different SA concentrations. (C) Effect of different BQ concentrations. Data are presented as mean ± SD (*n* = 3).

To elucidate the degradation mechanism and identify the primary oxidizing species in the MW-EP process, radical scavenging experiments were conducted using *tert*-butyl alcohol (TBA), *p*-benzoquinone (p-BQ), and sodium azide (NaN_3_). Initially, the effect of scavenger dosage was systematically investigated to ensure sufficient radical quenching without overdosing. As illustrated in Figure 10, increasing the scavenger concentration from 10 to 30 mM resulted in a progressive decline in the dye removal efficiency, indicating the effective interception of active species. However, increasing the dosage further to 50 mM yielded negligible changes, suggesting that the competitive reaction reached a saturation plateau at 30 mM. Consequently, this concentration was selected as the optimal dosage for mechanistic analysis.

**Figure 11:**
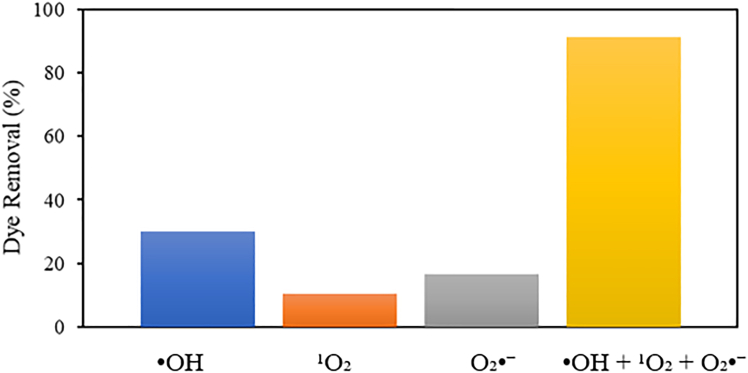
Comparison of reactive species in the process with the control experiment [Dye] = 300 mg/L, pH = 6.5, *p* = 279.04 W, I = 400 mA, [O_3_] = 0.288 g/h, [NaCl] = 0.035 M, and electrode distance (D) = 3 cm; [TBA] = [SA] = [BQ] = 30 mM.

Under these optimized conditions, the specific contribution of each radical species was evaluated against the control efficiency of 91.36% (Figure 11). The addition of TBA, a selective ȮH scavenger, caused a substantial drop in efficiency to 61.47%, confirming that ȮH radicals act as the dominant oxidizing species driving the degradation.^39^ Crucially, the presence of p-BQ also resulted in a significant reduction, lowering the efficiency to 74.89%. This distinct decrease demonstrates that superoxide radicals (O_2_⋅^−^) play a vital and active role in the reaction pathway and cannot be considered negligible.^40^ Furthermore, the introduction of NaN_3_ led to the sharpest decline (to 51.23%). Because NaN_3_ is known to scavenge both ȮH and singlet oxygen (^1^O_2_), this severe inhibition is largely attributed to the cumulative quenching of the dominant ȮH radicals along with a minor contribution from ^1^O_2_.^41^ Therefore, it can be concluded that the degradation of RY84 is primarily governed by the synergistic attack of ȮH and O_2_⋅^−^ radicals, rather than relying on a single oxidative pathway.

### Evaluation of COD and TOC removal under optimal conditions

**Figure 12:**
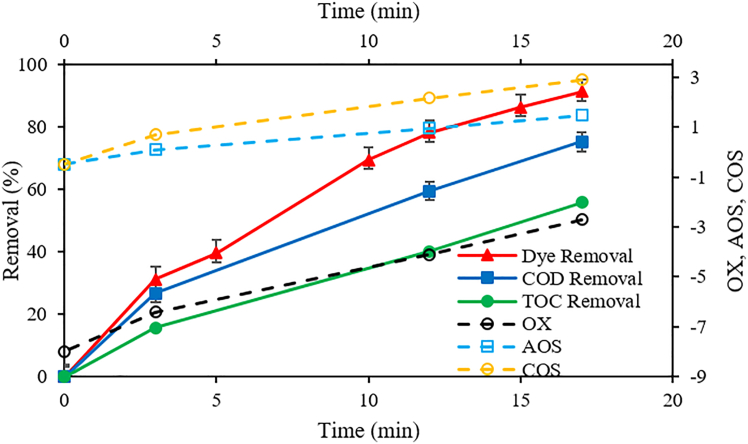
Removal efficiencies for TOC, COD, and dye, and variations of AOS and COS over time under optimal conditions [Dye] = 300 mg/L, pH = 6.5, *p* = 279.04 W, I = 400 mA, [O_3_] = 0.288 g/h, [NaCl] = 0.035 M, and electrode distance (D) = 3 cm. Data are presented as mean ± SD (*n* = 3).

After determining the optimal process conditions, changes in COD and TOC were monitored to evaluate the degradation mechanism and mineralization extent of the dye (Figure 12). The removal efficiencies for the dye, COD, and TOC after 17 min were 91.36%, 75.3%, and 55.8%, respectively. It is evident that the dye removal efficiency significantly surpasses those for COD and TOC. This distinct gap between decolorization and mineralization confirms that the degradation process occurs in stages: first, the rapid cleavage of the chromophoric azo bonds (–N=N–) leads to quick color disappearance; second, the resulting intermediate fragments—likely aromatic amines and phenolic compounds—require more energy and time to be fully mineralized into CO_2_ and H_2_O.

Although specific degradation intermediates were not identified via chromatography in this study, the discrepancy between dye and TOC removal clearly points to the formation of stable organic by-products during partial oxidation.^42^ To further analyze this transformation, oxidation indices were evaluated. The oxidation state (OX) results, as shown in Figure 12, indicate that the initial OX value increased from −8 to −2.7 during the process (with initial COD and TOC concentrations of 600 and 200 mg/L, respectively). The continuous increase in OX reflects the progression of oxidation, the breakdown of complex organic structures, and their gradual conversion into mineral compounds.^12^

Additionally, the average oxidation state (AOS) and carbon oxidation state (COS) values increased from −0.5 to 1.48 and 2.89, respectively. This upward trend indicates the removal of a major portion of the recalcitrant compounds and their transformation into more oxidized species such as carboxylic acids, aldehydes, and alcohols. From an environmental perspective, this shift is significant; while complete mineralization was not achieved, the substantial increase in OXs suggests a reduction in the toxicity of the effluent and an enhancement in its biodegradability, making the MW-EP-treated water more suitable for biological post-treatment or safe discharge compared with the raw textile wastewater. Similar findings regarding the correlation between oxidation indices and reduced toxicity have been reported previously.^43^

### Reaction kinetics

The kinetic analysis results based on zero-, first-, and second-order models, along with the corresponding reaction rate constants and correlation coefficients, are presented in Table 1. Data fitting indicated that the first-order model provided the best agreement with the experimental results. This finding suggests a direct dependence of the pollutant removal rate on its concentration in both the MW-EP and standalone systems. Mechanistically, the fit to first-order kinetics indicates the predominance of unimolecular reactions between the pollutant and reactive radicals such as ȮH and active oxygen species generated from O_3_, where the reaction rate is mainly governed by the probability of effective collisions between the radicals and pollutant molecules.^22^ The uniformity of the data distribution and minimal deviation from the fitted line validates this model in describing the MW-EP system behavior, indicating that the concentration of oxidizing active species remains approximately constant throughout the reaction.

**Table 1:** Reaction rate constants and correlation coefficients for the kinetic models

Process	Reaction order	Rate constant (k)	Correlation coefficient (R^2^)
Microwave (after 17 min, 9% removal)	zero-order	k_0_ = 1.82801	0.986
	first-order	k_1_ = 0.00568	0.988
	second-order	k_2_ = 0.0000177	0.987
Electro-peroxone (after 17 min, 66% removal)	zero-order	k_0_ = 11.7	0.982
	first-order	k_1_ = 0.0615	0.995
	second-order	k_2_ = 0.00035	0.956
Electro-peroxone (after 40 min, 92% removal)	zero-order	k_0_ = 7.45	0.934
	first-order	k_1_ = 0.0605	0.995
	second-order	k_2_ = 0.00071	0.843
Microwave-electro-peroxone (after 17 min, 91.36% removal)	zero-order	k_0_ = 16.16	0.950
	first-order	k_1_ = 0.12825	0.991
	second-order	k_2_ = 0.00135	0.923

### Reaction mechanism of MW-EP process

**Figure 13:**
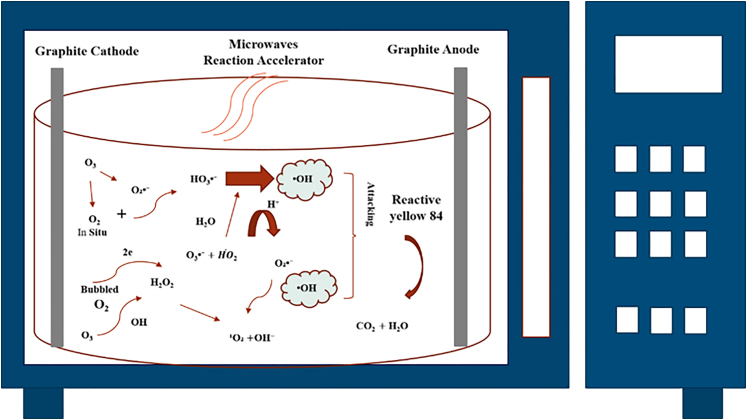
Schematic illustration of the MW-EP process mechanism

Based on the experimental results and the radical scavenger analysis, the probable reaction mechanism for the degradation of RY84 in the MW-EP system is schematically illustrated in Figure 13. The degradation process is initiated by the simultaneous generation of oxidants and the catalytic influence of MW irradiation.

First, the dissolved oxygen is reduced at the surface of the graphite cathode via a two-electron pathway to generate H_2_O_2_ *in situ* (Equation 11). Simultaneously, O_3_ is continuously injected into the reactor.

(Equation 11) O_2_ + 2H^+^ + 2e^−^ → H_2_O_2_

The electro-generated H_2_O_2_ dissociates into HO_2_^−^, which then react with O_3_ to initiate the peroxone reaction (Equations 12 and 13). This interaction is the primary source of ȮH and O_2_·^−^:

(Equation 12) H_2_O_2_ ⇌ HO_2_^−^ + H^+^

(Equation 13) O_3_ + HO_2_^−^ → ȮH + O_2_⋅^−^ + O_2_

A critical feature of this system is the role of MW irradiation. As shown in the mechanism, MWs do not merely heat the solution; they act as a powerful reaction accelerator. The MW energy is absorbed by the water molecules and polar intermediates, leading to rapid dipolar rotation and ionic conduction. This specific effect reduces the E_a_ of the peroxone reaction and enhances the cleavage of the O–O bond in O_3_ and peroxide molecules. Consequently, the production rate of ROS is significantly amplified compared with conventional conditions.

The radical chain propagation continues as O_2_⋅^−^ react with O_3_ to form ozonide radical anions (O_3_⋅^−^), which eventually decompose into more ȮH (Equations 14, 15, and 16). Additionally, ^1^O_2_ is generated as a selective oxidant through secondary reactions (Equation 17):

(Equation 14) O_3_ + O_2_⋅^−^ → O_3_⋅^−^ + O_2_

(Equation 15) O_3_⋅^−^ + H^+^ ⇌ HO_3_⋅

(Equation 16) HO_3_⋅ → ȮH + O_2_

(Equation 17) O_2_⋅^−^ + ȮH → ^1^O_2_ + OH^−^

Finally, the abundant ȮH radicals, energized by the MW field, non-selectively attack the azo dye structure. The continuous bombardment of the pollutant molecules results in the cleavage of azo bonds (–N=N–) and the breakdown of aromatic rings, ultimately leading to complete mineralization into carbon dioxide and water (Equation 18):

(Equation 18) RY84 + ȮH / MW → intermediates → CO_2_ + H_2_O + inorganic salts

### Energy consumption

Energy efficiency is a critical parameter for evaluating the industrial applicability of AOPs. In this study, the SEC was calculated based on the total power consumed by the MW irradiation, O_3_ generation, and electrochemical system.

As shown in Table 2, the MW-EP combined process demonstrated a remarkable reduction in energy requirements compared with the standalone EP process. The SEC for the MW-EP system was calculated at 579.25 kWh/kg, whereas the standalone EP process required 801.26 kWh/kg. This corresponds to a significant energy saving of 222.01 kWh/kg (approximately 27.7% reduction). This improvement is directly attributed to the synergistic effect, where the MW irradiation accelerates the reaction kinetics, drastically reducing the treatment time from 40 min (in EP) to 17 min (in MW-EP) for similar removal efficiencies (>91%).

**Table 2:** Comparative analysis of energy consumption and removal efficiency under various conditions

Method	Pollutant	Optimal conditions	Removal (%)	Energy consumption (kWh/kg)	Reference
Microwave/cobalt ion/peroxymonosulfate	[NH_4_^+^-N]	MW_power_ = 160 W, [Co^2+^] = 17.5 μM, [PMS] = 10 mM, [Cl] = 20 mM, [NH_4_^+^N] = 30 mg/L, 9 min	89.68	1626.72	Huang and Feng^44^
Heterogeneous electro-Fenton-like/MnCo_2_O_4_	paracetamol	adsorbent dose and catalyst dose = 1.2 g/L, [PCT] = 20 mg/L, room temperature, pH 7, [Na_2_SO_4_] = 0.05 mol/L, J = 25 mA/cm^2^, 90 min	99.07	434	Dhiss et al.^45^
Heterogeneous electro-Fenton/Fe-MIL-88B	Acid Blue 25	0.3 g/L Fe-MIL-88B, pH 3, [Dye] = 75 mg/L, I = 0.228 A, rotation velocity = 100 rpm, 90 min	92.3	1120.64	Najafzadeh and Ayati^46^
Electrochemical oxidation/Ti/PbO_2_ and Ti/BDD electrodes	carbamazepine	C = 10 mg/L [Na_2_SO_4_] = 400 mg/L, J = 100 mA/cm^2^ 20 min	88	4400	García-Gómez et al.^47^
Photoelectro-Fenton	triclopyr (TCP)	[Na_2_SO_4_] = 0.05 M, [TCP] = 0.12 mM, pH 7, 40 min	78	2820	Da Costa Soares et al.^48^
EP	Reactive Yellow 84	[Dye] = 300 mg/L, pH = 6.5, *p* = 279.04 W, I = 400 mA, [O_3_] = 0.288 g/h, [NaCl] = 0.035 M, 40 min	92	801.26	this study
MW-EP	Reactive Yellow 84	[Dye] = 300 mg/L, pH = 6.5, *p* = 279.04 W, I = 400 mA, [O_3_] = 0.288 g/h, [NaCl] = 0.035 M, 17 min	91.36	579.25	this study

To further benchmark the proposed method, the SEC of the MW-EP process was compared with those of other reported electrochemical and AOP methods (Table 2). While some heterogeneous processes, such as the electro-Fenton-like system using MnCo_2_O_4_, were reported to have lower energy consumption (∼434 kWh/kg),^45^ they required significantly longer reaction times (90 min) to achieve comparable removal. In contrast, the MW-EP process achieved high efficiency in a fraction of that time (17 min), implying a higher throughput and smaller reactor footprint for industrial upscaling. Furthermore, compared with other high-energy methods like electrochemical oxidation with boron-doped diamond (BDD) anodes (∼4,400 kWh/kg)^47^ or photoelectro-Fenton (∼2,820 kWh/kg),^48^ the MW-EP system proves to be a highly energy efficient alternative for the degradation of recalcitrant organic pollutants.

### Industrial usages

The primary objective of this study was to elucidate the fundamental degradation mechanism and optimize the operational parameters of the novel MW-EP system using synthetic wastewater. While the use of synthetic effluents allows for a precise understanding of reaction kinetics without the interference of complex scavenging agents found in real matrices, the transition to industrial applications remains the ultimate goal.

The MW-EP process demonstrates significant potential for industrial scalability due to three key factors as follows:

• Energy efficiency: As evidenced by the SEC analysis (Table 2), the MW-EP system achieves high degradation rates at a competitive energy cost compared with conventional incineration or single-process AOPs.
• Operational sustainability: Unlike homogeneous Fenton processes that generate large volumes of iron sludge requiring secondary disposal, the MW-EP system is a “clean” technology. The use of durable graphite electrodes (as discussed in Section 2.1 regarding reusability) eliminates the need for continuous chemical dosing and catalyst regeneration, making it operationally simpler for textile industries.
• Adaptability: The *in situ* generation of oxidants allows the system to be retrofitted into existing electrochemical treatment plants as a polishing step for recalcitrant dyes.

Therefore, while this study establishes the “proof-of-concept” in a controlled environment, the method is technically viable for treating real textile effluents, particularly those with high conductivity and refractory organic content.

## Discussion

The present study demonstrated that the MW-EP process is an efficient advanced oxidation technology for the degradation of RY84. The integration of MW irradiation with electrochemical H_2_O_2_ generation and O_3_ injection significantly enhanced the production of reactive oxidizing species, resulting in rapid pollutant removal. The MW-EP system achieved 91.36% removal within 17 min with lower energy consumption (579.25 kWh/kg) compared with the standalone EP process (92% in 40 min, 801.26 kWh/kg). The synergy coefficient (SE ≈ 1.91) confirms a strong interaction between MW irradiation and electrochemical oxidation.

From a practical standpoint, the MW-EP process shows strong potential for treating dye-containing industrial effluents, particularly as a rapid and energy-efficient pre- or post-treatment step. Nevertheless, further research is required to optimize key operational parameters, evaluate system performance in real wastewater matrices, and extend its applicability to other recalcitrant pollutants. In addition, future studies should investigate the formation and toxicity of intermediate by-products and conduct comprehensive techno-economic assessments under continuous flow conditions to validate large-scale applicability.

### Limitations of the study

This work was performed at the laboratory scale by using a single model dye solution; so, performance may differ in real industrial wastewater matrices. Optimization used a one-factor-at-a-time (OFAT) approach, which does not capture interactions between variables. Only dye removal, COD, and TOC were quantified, while by-products and toxicity changes were not fully assessed. Scale up, continuous operation, long-term electrode stability, and a complete techno-economic evaluation were not examined.

## Resource availability

### Lead contact

Requests for further information, resources, data, and reagents should be directed to and will be fulfilled by the lead contact, Bita Ayati (ayati_bi@modares.ac.ir).

### Materials availability

This study did not generate new unique reagents. All chemicals and materials used in this work are commercially available and listed in the key resources table. No materials require a material(s) transfer agreement (MTA).

### Data and code availability

• Data: All data supporting the findings of this study are available within the article and its supplemental information files.
• Code: This study did not generate any custom code.
• Other: Any additional information required to reanalyze the data reported in this paper is available from the corresponding author upon reasonable request.

## Acknowledgments

The authors gratefully acknowledge the laboratory facilities and technical assistance provided by Tarbiat Modares University. This research did not receive any specific grant.

## Author contributions

Conceptualization, investigation, methodology, resources, and writing – review & editing, S.M.R.M.M. and B.A.; writing – original draft, S.M.R.M.M.; supervision, B.A.

## Declaration of interests

The authors declare no competing interests.

## STAR★Methods

### Key resources table

**Table:** 

REAGENT or RESOURCE	SOURCE	IDENTIFIER
**Chemicals, peptides, and recombinant proteins**		

Reactive Yellow 84 (RY84) dye	Sigma-Aldrich	CAS: 61951-85-7
Sodium chloride (NaCl)	Merck	CAS: 7647-14-5
Caustic soda (NaOH)	Merck	CAS: 1310-73-2
Hydrochloric acid (HCl)	Merck	CAS: 7647-01-0
Sodium sulfate (Na_2_SO_4_)	Merck	CAS: 7757-82-6
Sodium phosphate (Na_3_PO_4_)	Merck	CAS: 7601-54-9
Sodium propionate (C_3_H_5_NaO_2_)	Merck	CAS: 137-40-6
tert-Butanol (TBA)	Merck	CAS: 75-65-0
Sodium azide (SA)	Merck	CAS: 26628-22-8
p-Benzoquinone (p-BQ)	Merck	CAS: 106-51-4
Distilled water	Laboratory supply	N/A

**Critical commercial assays**		

COD reagent kits	Hach	Code: K0012
Hach TOC reagent kits	Hach	Code: K031

**Other**		

DC power supply (PM-3005D)	Megatek	Model PM-3005D
Microwave oven (M245)	Butane Co., Iran	Model M245
UV–Vis spectrophotometer (DR 6000)	Hach, USA	Model DR 6000
Digital pH meter (691)	Metrohm	Model 691
Digital balance (PJ300)	Mettler, Switzerland	Model PJ300
Ozone generator (Arda Cog 5s)	Arda	Model Cog 5s
Ozone analyzer (968)	BMT	Model 968
Rotameter (AZR-100)	Abzar Daghigh	Model AZR-100
COD reactor (DRB200)	Hach	Model DRB200
Mechanical stirrer (RW 20)	IKA	Model RW 20

### Method details

#### Experimental setup

It is important to emphasize that this study employed synthetic wastewater with a fixed composition (RY84 dissolved in distilled water) rather than real textile effluent. While real wastewater is characterized by complex and fluctuating matrices (varying over time and production cycles), the use of a synthetic solution in this work was a strategic choice to isolate the specific degradation mechanisms of the MW-EP process. This controlled environment ensures reproducibility and allows for a precise kinetic evaluation without the interference of unknown scavengers or variable auxiliary chemicals.

A one-liter glass reactor was employed for the MW–EP process. The working volume of 1.0 L was selected to ensure hydrodynamic stability and minimize kinetic errors caused by solution volume reduction during periodic sampling, while also serving as a representative bench-scale setup to provide accurate energy consumption data for potential scale up considerations.^49^ Glass was specifically selected as the reactor material due to its transparency to microwave irradiation, ensuring maximum energy penetration into the solution without the shielding effects associated with metallic reactors.^50^ Two graphite electrodes with an effective surface area of 181 cm^2^ (dimensions: 16 × 0.5 × 5 cm) were installed inside the reactor and connected to a DC power supply. Although advanced electrode materials such as Boron-Doped Diamond (BDD) or Gas Diffusion Electrodes (GDE) often exhibit superior oxidative capacity or H_2_O_2_ generation rates, graphite was chosen in this study to optimize the cost-performance ratio. Graphite provides a practical balance between electrochemical efficiency and economic feasibility compared to high-cost alternatives like BDD or MMO, making the process more attractive for potential industrial scaling.^51^ Oxygen gas was supplied via a cylinder connected to a rotameter, which was further linked to an ozone generator to produce the ozone required for the process. Microwave irradiation was applied using a microwave device. Additionally, a mechanical stirrer was incorporated to ensure uniform mixing within the reaction medium. A schematic of the experimental setup is presented in below figure.

**Figure:**
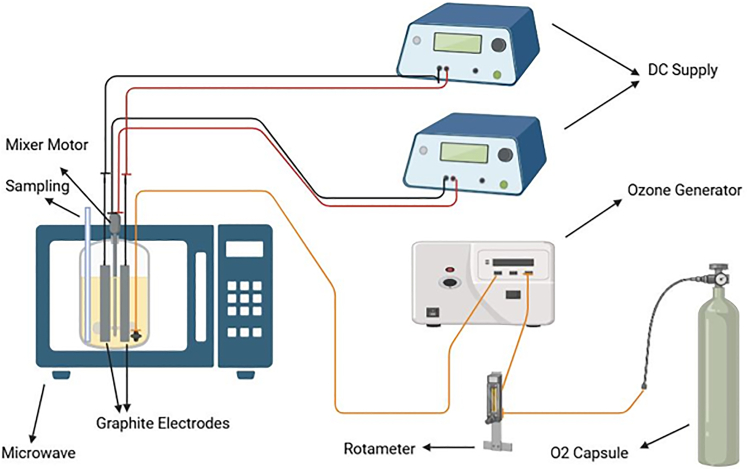
Schematic of the MW-EP process used in this study

#### Experimental procedure

In this study, the optimization was conducted using the One-Factor-At-a-Time (OFAT) approach. This method was selected to explicitly identify the dominant individual factors governing the kinetics of the novel MW-EP hybrid system. While OFAT effectively screens critical variables, we acknowledge that it does not quantify parameter interactions as comprehensively as multivariate statistical methods. Recent studies have successfully employed designs such as Full Factorial,^52^ Box–Behnken,^53^ and Central Composite Design^54^ to model such interactions. However, given the exploratory nature of this work, OFAT provided a direct evaluation of the system’s baseline performance, paving the way for future multivariate optimization.

The operational parameters and their specific ranges were established based on a synthesis of previous studies,^22^^,^^55^^,^^56^ preliminary screening experiments, and the practical limits of the available facilities. A summary of these investigated variables is presented in below table.

**Table:** Investigated parameters

Parameter	Values
Initial pollutant concentration (mg/L)	150, 200, 300, 400, and 500
pH	3, 6.5, and 9
Microwave power (W)	161.32, 279.04, 354.27, and 527.37
Current intensity (mA)	200, 400, 600, and 800
Ozone flow rate (g/h)	0.078, 0.171, 0.271, and 0.288
Electrolyte type	NaCl, Na_2_SO_4_, Na_3_PO_4_, and C_3_H_5_NaO_2_
Electrolyte concentration (M)	0.02, 0.035, 0.05, 0.1, and 0.2
Inter-electrode distance (cm)	3, 5, and 7
Reaction time (min)	0–17

First, the initial pollutant concentration and pH ranges were chosen to simulate the variable conditions typical of real textile wastewater, allowing for an evaluation of the system’s robustness under both acidic and alkaline environments. The electrolyte concentration and types were varied to investigate their dual role in enhancing solution conductivity and their potential scavenging effects on radical species. Second, regarding energy input, the applied current and microwave power were adjusted to identify the optimal trade-off between degradation kinetics and energy consumption (cost-effectiveness). The ozone flow rate was limited to 0.078–0.288 g/h to minimize the mass transfer resistance while preventing the wastage of unreacted ozone. Finally, the inter-electrode distance was investigated to minimize the energy loss. The reaction time was strictly capped at 17 min to prevent the solution temperature from reaching the boiling point under microwave irradiation, thereby maintaining process stability and safety.

The monoazo dye Reactive Yellow 84, with the chemical formula C_50_H_24_Cl_2_N_14_Na_10_O_30_S_10_ and a molecular weight of 1628.22 g/mol, and an initial pH of 6.5, was selected as the model pollutant in this study. To determine the dye concentration, a calibration curve was constructed using standard solutions with concentrations of 10, 20, 50, 100, 150, 200, 300, 400, and 500 mg/L. The absorbance of these standard solutions was measured spectrophotometrically at the maximum absorption wavelength of 418 nm.

All experiments were conducted at room temperature (25 ± 1 °C) in triplicate, with a 5% error margin. Since the microwave device used allowed power adjustment only as a percentage of the nominal 800 W, the actual output power at different levels was experimentally measured and corrected. Accordingly, the power values applied in the experiments were based on the actual power delivered to the system (rather than the nominal percentage) and were used in all calculations and analyses to improve the accuracy of evaluating the effect of microwave power on process efficiency. Additionally, due to water boiling inside the device and the effect of bubbles generated during boiling on the reaction, the reaction time was set up to the point of boiling. A calibration curve correlating nominal microwave power settings with actual power output was constructed (Figure S1). Figure S2 shows the boiling point of water at the corresponding power levels.

In the electro-peroxone process, determining the ozone output in terms of mass flow rate (g/h) is of critical importance. Expressing ozone in mass flow rate allows for precise control and monitoring of the actual ozone production or injection rate into the system. Since ozone is typically introduced into the pilot system as a gas, a portion dissolves in water while the remainder either does not participate in the reaction or escapes. However, to minimize this loss and maximize gas utilization, the reactor in this study was designed with a semi-closed configuration (venting restricted to the stirrer shaft), forcing the ozone to remain in the headspace until dissolution.

Therefore, expressing the injected ozone solely in terms of solution concentration (e.g., mg/L) does not accurately reflect the actual ozone consumption or process efficiency, especially since direct monitoring of dissolved ozone was precluded by electromagnetic interference inside the microwave cavity. In contrast, using g/h provides a basis for design calculations, energy consumption assessment, and comparison of process performance at laboratory and industrial scales.^57^

The output ozone mass flow rate in g/h was obtained by multiplying the input ozone concentration in mg/L (measured via a UV-photometric ozone analyzer) by the oxygen flow rate in mL/min. Below table presents the results of the calibration of the rotameter and ozone generator. Due to equipment limitations, calibration of the rotameter for ozone flow rates above 0.288 g/h was not possible.

**Table:** Results of the calibration of the rotameter and ozone generator

Rotameter reading	Ozone input (mg/L)	Oxygen flow rate (mL/min)	Ozone injection rate (g/h)
28	26	50	0.078
32	24	100	0.144
36	19	150	0.171
40	18.3	200	0.2196
52	13	300	0.234
62	11.3	400	0.271
76	8.7	500	0.261
85	8	600	0.288

To investigate the specific role of reactive species in the removal of Reactive Yellow 84, tert-butanol (TBA) was used as a specific hydroxyl radical (ȮH) scavenger,^39^ sodium azide (SA), which not only inhibits hydroxyl radicals but also deactivates singlet oxygen (^1^O_2_),^40^ and p-benzoquinone (p-BQ) as a superoxide radical (Ȯ_2_^−^) scavenger^41^ under the optimal conditions.

#### Materials and analytical instruments

Reactive Yellow 84 dye was purchased from Sigma-Aldrich. Sodium chloride (NaCl) was used as the electrolyte to enhance the electrical conductivity of the solution. The pH of the solutions was adjusted using caustic soda (NaOH) and hydrochloric acid (HCl), both supplied by Merck. To investigate the optimal type of electrolyte, sodium sulfate (Na_2_SO_4_), sodium phosphate (Na_3_PO_4_), and sodium propionate (C_3_H_5_NaO_2_) were employed. For the identification of reactive species in the process, tert-butanol (TBA), sodium azide (SA), and p-benzoquinone (p-BQ), all supplied by Merck, were used. Chemical oxygen demand (COD) analysis was performed using specific kits (code: K0012), and total organic carbon (TOC) analysis was conducted using Hach reagent kits (code: K031). Distilled water was used as the solvent in all experiments.

A direct current (DC) power supply (Megatek, PM-3005D) was used to provide the electrical current. Microwave irradiation was applied using a microwave device (Butane Co., Iran, model M245) with a maximum nominal power of 800 W. Dye concentration as well as COD and TOC were measured using a spectrophotometer (Hach, USA, model DR 6000). The pH of the solutions was measured with a digital pH meter (Metrohm, model 691) equipped with a digital electrode. For precise weighing of samples, a digital balance (Mettler, Switzerland, model PJ300) with an accuracy of 0.001 g was used. Ozone injection was performed using an ozone generator (Arda Cog 5s), and the output ozone concentration was determined with an analyzer (BMT, model 968). Oxygen was supplied to the ozone generator using a glass-body rotameter (Abzar Daghigh, model AZR-100). COD analysis was also conducted using a COD reactor (Hach, model DRB200). For mixing, a mechanical stirrer (IKA RW 20) was employed.

#### Calculations

Linear regression analysis was performed on the absorbance versus concentration data, yielding a calibration coefficient (slope) of 0.0176. Consequently, the unknown dye concentration in the samples was calculated using the following Equation 19 based on the Beer-Lambert law, where C is expressed in mg/L.

(Equation 19) $$C=\frac{\text{Abs}}{0/0176}$$

To evaluate the dye removal efficiency at different time intervals, Equation 20 was used, where C_0_ is the initial dye concentration, C_t_ is the dye concentration at time t, and R represents the percentage of pollutant removal at time t.

(Equation 20) $$R=\left|\frac{C_{t}-C_{0}}{C_{0}}\right|\times 100$$

Finally, the probable intermediate compounds were investigated under optimal conditions by measuring COD, TOC, and oxidation state according to Equation 21.^9^

(Equation 21) $$\text{Oxidation}\;\text{State}\;\left(\text{OX}\right)=\frac{4\left(\text{TOC}-\text{COD}\right)\;}{\text{TOC}}$$

Furthermore, the improvement in the biodegradability of the wastewater was evaluated using biodegradability assessment indices, including the carbon oxidation state (COS) and the average oxidation state (AOS). The AOS and COS values range from –4 to +4, representing the most reduced state (methane) and the most oxidized state (carbon dioxide) of carbon, respectively. The AOS and COS values were calculated based on Equations 22 and 23.^58^

(Equation 22) $$\text{Average}\;\text{Oxidation}\;\text{State}\;\left(\text{AOS}\right)=4-\;\frac{1.5\;\text{COD}\;}{\text{TOC}}$$

(Equation 23) $$\text{Carbon}\;\text{Oxidation}\;\text{State}\;\left(\mathrm{COS}\right)=4-\;\frac{1.5\;\text{COD}\;}{\text{TOC}0}$$

where COD is the chemical oxygen demand (mg/L), TOC is the total organic carbon (mg/L), and TOC_0_ is the initial total organic carbon concentration (mg/L).

In the process under investigation, due to the use of electricity as the energy source, the energy consumption is of particular importance for determining the optimal operating conditions. The energy consumption of the electro-peroxone process (kWh/kg) was calculated using Equation 24, where U is the voltage (V), I is the current intensity (A), V is solution volume (L), t is the reaction time (h), and C_0_ is the initial pollutant concentration (mg/L).

(Equation 24) $$\text{Energy}\;\text{Consumption}\;\left(\text{EP}\right)=\frac{U\times I\times t\;}{\;V{\times C}_{0\;}\times 10^{-3}}$$

Since ozone production using the ozone generator in the ozonation process also requires electrical energy, the energy consumed for ozone generation was calculated based on the power consumption of the ozone generator, which was 0.035 kW according to the device specifications. Additionally, the energy consumption of the electric stirrer was determined based on its power rating, which was 0.06 kW.

The energy consumption of the microwave device was also calculated using Equation 25, where

P is the power (kW), t is the reaction time (h), and C_0_ is the initial pollutant concentration (mg/L).

(Equation 25) $$\text{Energy}\;\text{Consumption}\;\left(\text{MW}\right)=\frac{P\times t\;}{\;V{\times C}_{0\;}\times 10^{-3}}$$

The total energy consumption was calculated by summing the energies computed from Equations 24 and 25 together with the fixed energy consumption of the ozone generator (0.06 kW) and the stirrer (0.035 kW), (Equation 26).

(Equation 26) $$\text{Energy}\;\text{Consumption}\;\left(\text{MW}\right)=\frac{\left(P\times t\right)+\left(U\times I\times t\right)+\left(P_{\text{Ozone}}+P_{\text{Stirrer}}\right)\times t}{V{\times C}_{0\;}\times 10^{-3}}$$

To investigate the effect of the combined simultaneous processes, the synergistic effect (SE) between EP and MW was calculated according to Equation 27.^59^

(Equation 27) $$\text{Synergistic}\;\text{Effect}=\frac{k_{\text{MW}-\text{EP}}\;}{\;k_{\text{EP}}+k_{\text{MW}}\;}$$

In the above equation, k_MW–EP_ is the rate constant of the combined process, k_EP_ is the rate constant of the electro-peroxone process, and k_MW_ is the rate constant of the microwave process, all expressed in 1/min.

Selecting an appropriate kinetic model is a key factor in understanding the mechanism of advanced oxidation reactions. To determine the suitable kinetic model for dye removal in the microwave–electro-peroxone system and to calculate the apparent reaction rate constant, zero-order, first-order, and second-order kinetic models were employed, with the corresponding equations presented in Equations 28, 29, and 30, respectively.

(Equation 28) $$C_{t}-C_{0}=k_{0}t$$

(Equation 29) $$\ln\frac{C_{0}}{C_{t}}=k_{1}t$$

(Equation 30) $$\frac{1}{C_{t}}-\frac{1}{C_{0}}=k_{2}t$$

In the above equations, t is the time in minutes, C_0_ is the initial dye concentration, C_t_ is the dye concentration at time t (mg/L), and k_0_, k_1_, and k_2_ are the rate constants for zero-, first-, and second-order reactions, respectively.^60^

### Quantification and statistical analysis

All experiments were conducted in triplicate, and the average values were used for data analysis. The experimental error was maintained within ±5%. Linear regression analysis was performed on the absorbance versus concentration data to obtain the calibration curve for Reactive Yellow 84. Apparent rate constants were estimated by fitting the experimental data to zero-order, first-order, and second-order kinetic models, and the goodness of fit was evaluated based on the correlation coefficient (R^2^).

### Additional resources

No additional resources were generated or analyzed in this study.
