# Supplementary material for: Microwave-assisted electro-peroxone process for rapid removal of high-concentration recalcitrant dye solutions
Source: iScience. 2026 Jul 20;29(8):116810. doi: 10.1016/j.isci.2026.116810 (PMC13393832; doi:10.1016/j.isci.2026.116810)
Supplement: Document S1. Figures S1 and S2 [file mmc1.pdf]

iScience, Volume *29*

Supplemental information

Microwave-assisted electro-peroxone process for rapid removal of high-concentration recalcitrant dye solutions

Seyed Masoud Razavi Mozafar Moghadam and Bita Ayati

Microwave conditions

A calibration curve relating the output power to the corresponding Wattage was plotted (Figure S1).

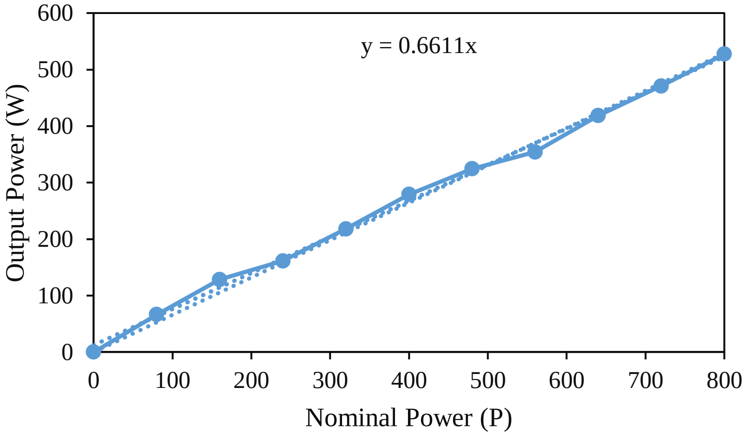

Figure S1. Calibration curve of nominal microwave power settings versus actual power output (W), related to the experimental procedure in the main text.

Figure S2 shows the boiling point of water at different microwave power levels. As observed, increasing the microwave power decreases the time required for the water to reach its boiling point.

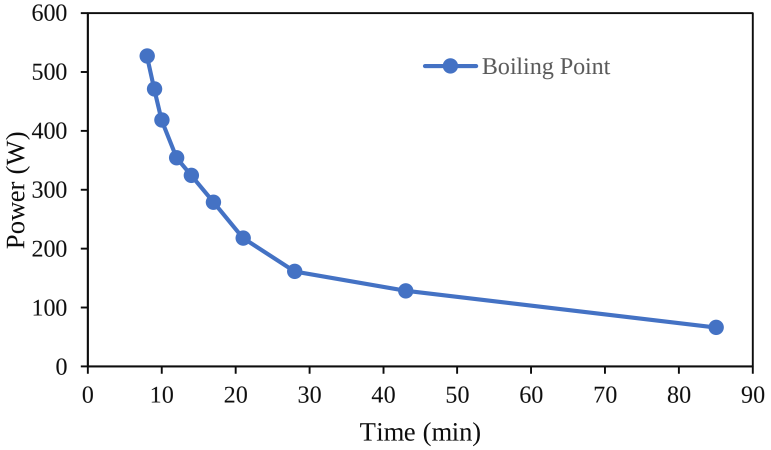

Figure S2. Boiling point of water at different microwave power levels, related to the experimental procedure in the main text.

Calculations of Synergistic Effect

$$\text{Synergistic Effect} = \frac{k_{MW\text{-}EP}}{k_{EP}+k_{MW}}$$

$$\text{Synergistic Effect} = \frac{0.12825}{0.0615+0.00568} = 1.90905$$
